# Identification of a Major QTL for Seed Protein Content in Cultivated Peanut (*Arachis hypogaea* L.) Using QTL-Seq

**DOI:** 10.3390/plants13172368

**Published:** 2024-08-25

**Authors:** Hao Chen, Nian Liu, Li Huang, Dongxin Huai, Rirong Xu, Xiangyu Chen, Shengyao Guo, Jianhong Chen, Huifang Jiang

**Affiliations:** 1 Institute of Crop Sciences, Fujian Research Station of Crop Gene Resource & Germplasm Enhancement, Ministry of Agriculture and Rural Affairs of People’s Republic of China, Fujian Engineering Research Center for Characteristic Upland Crops Breeding, Fujian Engineering Laboratory of Crop Molecular Breeding, Fujian Academy of Agricultural Sciences, Fuzhou 350013, China; bullnet_1985@163.com (H.C.);; 2 Oil Crops Research Institute of the Chinese Academy of Agricultural Sciences, Key Laboratory of Biology and Genetic Improvement of Oil Crops, Ministry of Agriculture and Rural Affairs of People’s Republic of China, Wuhan 430062, China; 3 Quanzhou Institute of Agricultural Sciences, Jinjiang 362212, China

**Keywords:** QTL-seq, BSA mapping, seed protein content, peanut

## Abstract

Peanut (*Arachis hypogaea* L.) is a great plant protein source for human diet since it has high protein content in the kernel. Therefore, seed protein content (SPC) is considered a major agronomic and quality trait in peanut breeding. However, few genetic loci underlying SPC have been identified in peanuts, and the underlying regulatory mechanisms remain unknown, limiting the effectiveness of breeding for high-SPC peanut varieties. In this study, a major QTL (*qSPCB10.1*) controlling peanut SPC was identified within a 2.3 Mb interval in chromosome B10 by QTL-seq using a recombinant inbred line population derived from parental lines with high and low SPCs, respectively. Sequence comparison, transcriptomic analysis, and annotation analysis of the *qSPCB10.1* locus were performed. Six differentially expressed genes with sequence variations between two parents were identified as candidate genes underlying *qSPCB10.1*. Further locus interaction analysis revealed that *qSPCB10.1* could not affect the seed oil accumulation unless *qOCA08.1_XH13_* was present, a high seed oil content (SOC) allele for a major QTL underlying SOC. In summary, our study provides a basis for future investigation of the genetic basis of seed protein accumulation and facilitates marker-assisted selection for developing high-SPC peanut genotypes.

## 1. Introduction

Cultivated peanut (*Arachis hypogaea* L.) is rich in oil and is known as a protein-rich crop, with an average of 25.8% crude protein in their seeds [1]. It provides a great economical source of easily absorbed, plant-based protein with possible cardiovascular-health-promoting functions, especially for those suffering from malnutrition in developing and low-income countries and regions [2]. Furthermore, peanut proteins also serve as the precursor to a variety of amino acids and bioactive peptides, which determine peanut products’ flavor and nutritional quality [3]. Hence, breeding peanut varieties with high seed protein content (SPC) is a promising approach for overcoming malnutrition and enhancing the nutritional and culinary value of peanut-based food products.

The SPC of cultivated peanut ranges from approximately 22 to 36%, depending on the variety [4]. Uncovering the genetic mechanisms underlying the differences in peanut SPC among different genotypes will assist in breeding high-protein peanut varieties. However, the genetic mechanism for protein accumulation in peanut seeds remains elusive. According to previous studies in peanut and other crops, SPC is a quantitative trait controlled by various genetic loci [5,6]. Moreover, peanut SPC is generally negatively correlated with oil content [7]. These results underline the complexity of the genetic basis for seed protein accumulation in peanut and the difficulty of breeding high-SPC peanut varieties. The recent advances in *Arachis* genomics enable the dissection of QTLs/genes underlying seed protein content through biparental mapping and genome-wide association studies. For example, Sarvamangala et al. identified eight QTLs underlying seed protein content from an RIL population consisting of 146 lines [8]. Sun et al. identified 29 QTLs controlling SPC in four environments using another RIL population of 318 lines [9]. Zhang et al. detected 22 significant QTLs associated with SPC through genome-wide association analysis on the U.S. peanut mini-core collection comprising 120 peanut accessions [10]. However, only a few QTLs/genes controlling SPC have been identified in peanut compared with those that control oil content. In order to select peanut lines/varieties carrying favorable QTLs/genes for higher protein content, more information about QTLs/genes is urgently required, especially for QTLs/genes with large effects and trait-specific characteristics.

In this study, a major QTL underlying SPC was identified using a QTL-seq approach. Potential candidate genes were assessed by analyzing the sequence and transcriptomic information in the mapping region. Its interaction relationship in seed protein/oil accumulation with *qOCA08.1*, a previously reported major QTL that controls oil content, was compared and evaluated.

## 2. Results

### 2.1. Phenotypic Variation for SPC in the RIL Population and Its Parental Lines

**Figure 1:**
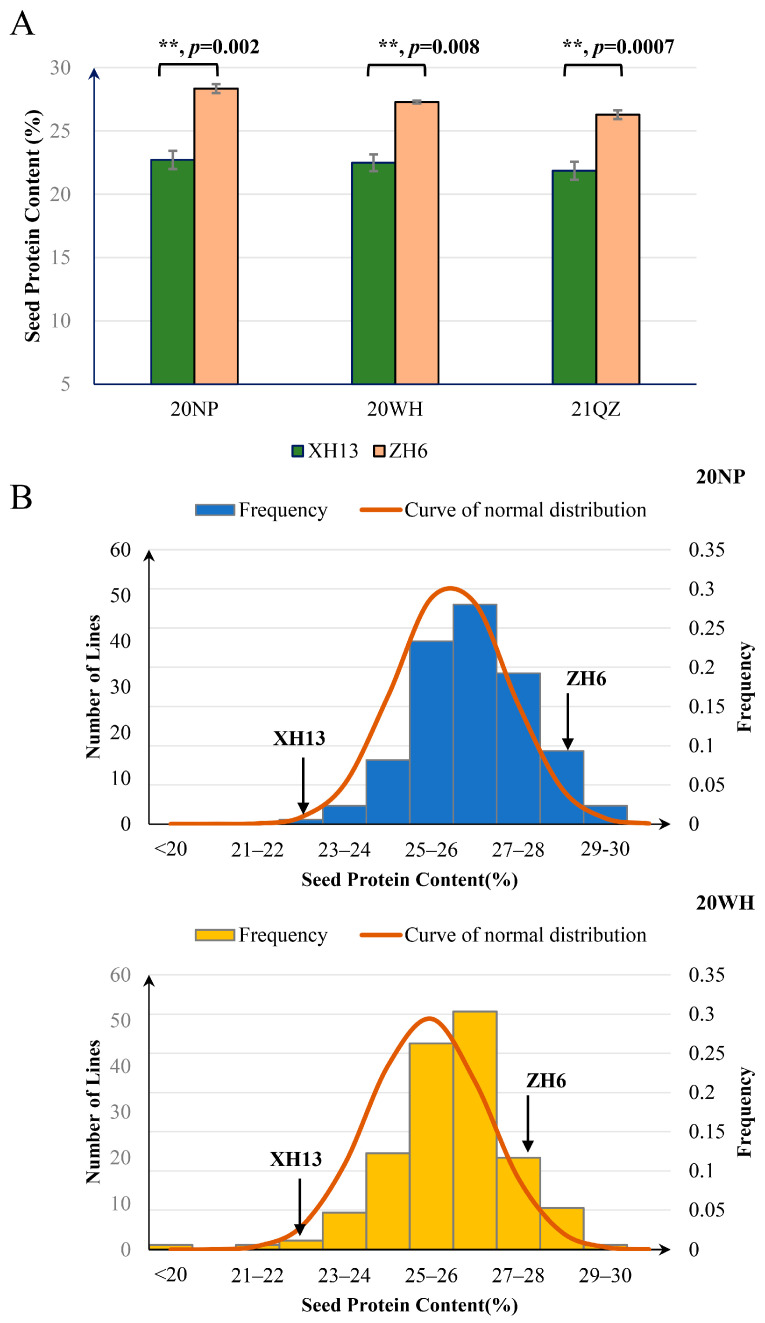
Phenotypic variation for SPC in the RIL population and its two parental lines. (**A**) SPC values in the parental lines ZH6 and XH13. Error bars represent the SE values; statistical significance was determined by unpaired *t*-tests; “**” represents *p* < 0.01. 20NP, 20WH, and 21QZ represent the data collected from Nanping City in the year 2020, Wuhan City in the year 2020, and Quanzhou City in the year of 2021, respectively. (**B**) Frequency distribution of SPC in RIL lines in 20NP and 20WH. The arrows indicate the SPC in XH13 and ZH6.

We measured the SPC of peanut cultivars “Zhonghua 6” (ZH6) and “Xuhua 13” (XH13), two elite peanut cultivars from China. ZH6 had a significantly higher SPC than XH13 in both tested environments, suggesting that the higher SPC in ZH6 can be stably inherited (Figure 1A). We further measured the SPC in an RIL population consisting of 160 lines derived from a cross between ZH6 and XH13. As a result, significant phenotypic variation for SPC was observed among the RILs, and a continuous distribution with transgressive segregation for SPC was also observed in the tested population across different environments (Figure 1B).

### 2.2. QTL-Seq Identification of a QTL Region Controlling SPC

**Figure 2:**
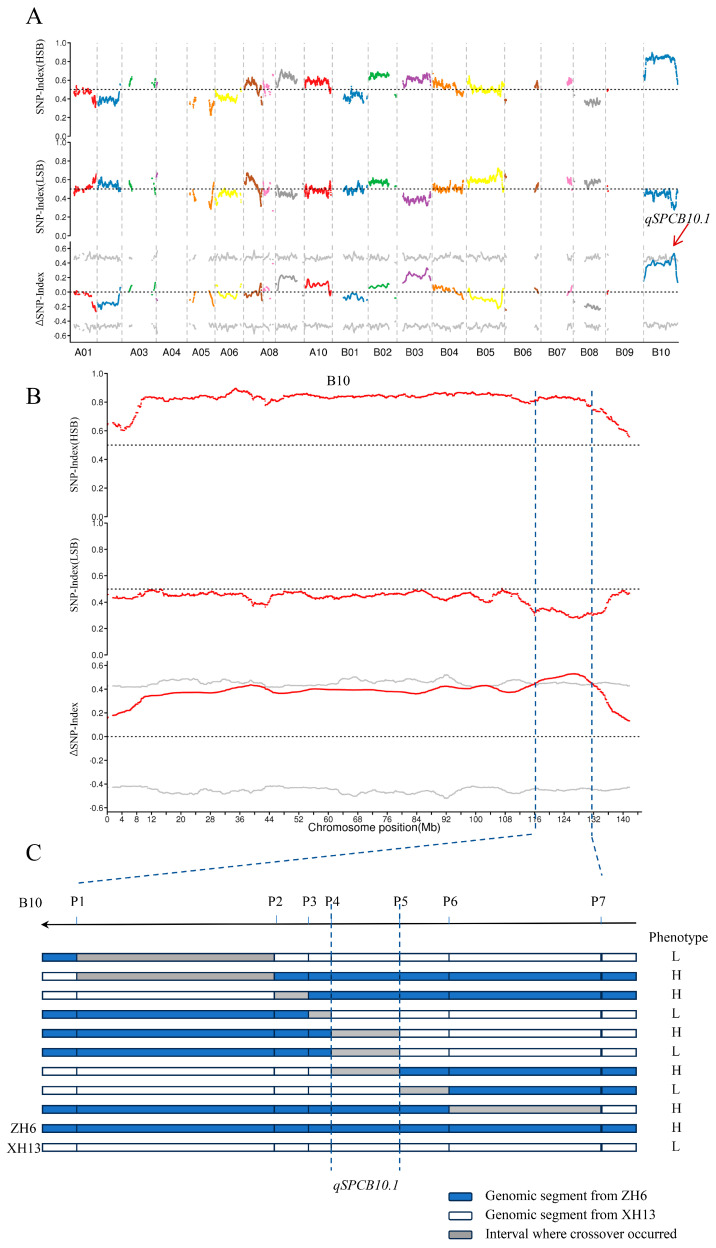
Mapping of the genomic region associated with SPC by QTL-seq. (**A**) Distribution of genome-wide single nucleotide polymorphisms between high-SPC and low-SPC pools. (**B**) ΔSNP index distribution across chromosomes and the significant candidate interval of Δ (SNP index) on chromosome B10. (**C**) Narrowing down the mapping region of *qSPCB10.1* by substitution mapping. “H” represents lines with a high-SPC phenotype, while “L” represents lines with a low SPC.

In order to identify genomic regions associated with SPC, QTL-seq analysis was conducted. Based on the phenotypes from two trial sites, 25 lines from the RIL population with lower SPC values (all these lines exhibited SPCs of less than 25.50%) and 25 lines with higher SPC values (all these lines exhibited SPCs higher than 27%) were selected to construct two extreme bulks, the low-SPC bulk (LSB) and high-SPC bulk (HSB). These bulks were subsequently used for genomic sequencing. The genomic sequencing of LSB and HSB generated a total of 70,441,775 and 68,255,801 clean reads, respectively. The reads of the LSB achieved a 29.10-fold read depth of the cultivated peanut reference genome, while those from HSB reached a 28.51-fold depth. Meanwhile, the parental lines were also re-sequenced. After aligning these clean reads to the reference genome and performing variant calling, a total of 306,147 variants were identified from the two bulks and their parents. We determined the physical positions of SNPs and InDels in the cultivated peanut genome (Figure 2A). The SNP index for each identified SNP in LSB and HSB was calculated. The corresponding ΔSNP index between two bulks was subsequently obtained by subtracting the SNP index of LSB from the SNP index of HSB. Based on sliding window analysis of the SNP index and ΔSNP index plots, genomic regions with an SNP index that significantly deviated from 0.5 and an ΔSNP index that significantly deviated from zero were considered as candidate QTL regions controlling SPC. Based on these criteria, only one major peak located on chromosome B10 was identified for SPC, based on the 99% statistical confidence intervals (permutation tests under the null hypothesis of no QTLs). This major QTL, named as *qSPCB10.1*, was mapped in a genomic region within a 16.05 Mb physical distance (117.14–133.20 Mb on chromosome B10) (Figure 2B).

To narrow down the mapping region of *qSPCB10.1*, eight SNP markers, evenly distributed on the map region, were developed and implemented for genotyping the RILs. A total of nine RILs that exhibited recombination within *qSPCB10.1* mapping region were identified. Based on the genotypic and phenotypic data for SPC from these identified RIL lines, the *qSPCB10.1* locus was further delimited to a ~2.3 Mb genomic region between markers P4 and P5 (Figure 2C).

### 2.3. Gene Candidate Analysis for qSPCB10.1

According to the genomic annotation information, there were 63 predicted genes in the mapping region of *qSPCB10.1* (Table S1). At the same time, a total of 494 variants (410 SNPs and 84 InDels) between ZH6 and XH13 were identified in this interval (Tables S2 and S3). Functional annotation analysis of these variants revealed that 20 SNPs were located on the gene exons and resulted in 12 non-synonymous substitutions in two predicted genes, *arahy.3RFM2L* and *arahy.FQN6EF*, and eight synonymous substitutions in five predicted genes, *arahy.3RFM2L*, *arahy.FQN6EF*, *arahy.59G37Z*, *arahy.P2VYVD*, and *arahy.39ZYJP*, respectively. However, none of these variants caused large functional or structural variations (such as frame-shift, stop-gain/loss, or splicing) in those predicted genes described above. In addition, one SNP and four InDels were located in introns, and two SNPs and nineteen InDels were annotated in upstream/downstream of genes or at the UTR region. The remaining variants were located in intergenic regions of the genome.

**Figure 3:**
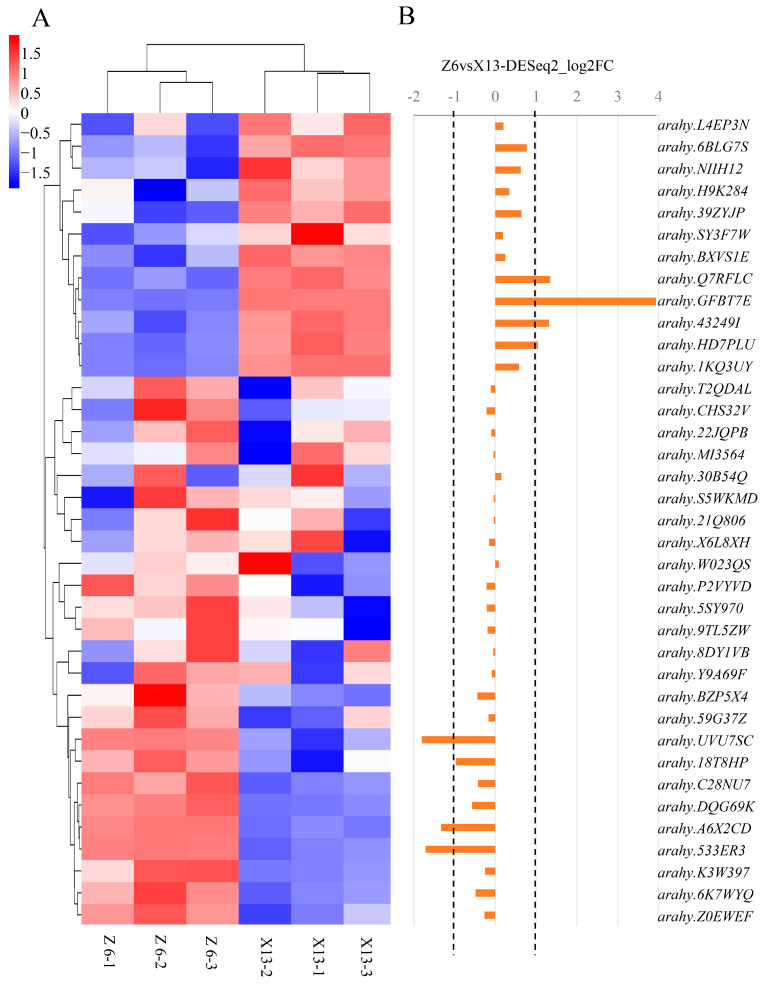
Transcriptomic data for the predicted genes in *qSPCB10.1* mapping region. (**A**) Heat map of the expression profiles of all expressed genes in *qSPCB10.1* mapping region in the developing seeds of two parental lines. (**B**) Expression profile comparisons of all expressed genes of *qSPCB10.1* mapping region in the developing seeds of two parental lines. The vertical axis represents the degree of differential gene expression (log two −fold change [log2FC]) of individual genes in *qSPCB10.1* mapping region. The dotted lines represent the threshold for identifying differentially expressed genes in this study.

A transcriptomic analysis was subsequently conducted to further investigate the expression profiles of putative candidate genes for *qSPCB10.1* using the developing kernels of ZH6 and XH13. As a result, 37 of 63 predicted genes in *qSPCB10.1* mapping region were found to be expressed above a threshold (FPKM > 0.5) (Figure 3A). Among these expressed predicted genes, seven genes exhibited significantly different expression levels (fold change (FC) ≥ 2 and false discovery rate (FDR) ≤ 0.05) between ZH6 and Xuhua 13 and were considered as differentially expressed genes (DEGs) (Figure 3B). Four exhibited higher expression levels in ZH6 than in XH13, while the rest of the DEGs had the opposite expression trend, being higher in XH13.

Combined with the sequence analysis data and gene expression profiling, it was found that *arahy.3RFM2L* and *arahy.FQN6EF*, two predicted genes that harbored synonymous and non-synonymous variants, did not exhibit any apparent expression. The gene expression for the other three predicted genes, *arahy.59G37Z*, *arahy.P2VYVD*, and *arahy.39ZYJP*, which harbored synonymous variants between the two parental lines, had detectable gene expression in the transcriptomic analysis. However, their gene expression levels did not significantly differ between ZH6 and XH13. These results suggested that those genes described above may not be the candidate genes for *qSPCB10.1*. We further analyzed the seven detected DEGs in the *qSPCB10.1* mapping region. Variants were found in the gene body or its surrounding region in six DEGs, with *arahy.533ER3* being the exception. The genes with both differential expression and sequence variations were *arahy.A6X2CD*, encoding a serine/threonine protein kinase; *arahy.UVU7SC*, encoding an AIG2-like protein; *arahy.Q7RFLC*, encoding a probable nucleoredoxin; *arahy.GFBT7E*, encoding an oleosin; *arahy.HD7PLU*, encoding a DEAD-box ATP-dependent RNA helicase; and *arahy.43249I*, encoding a hypothetical protein. We hypothesized that these genes were the possible candidates for *qSPCB10.1*.

### 2.4. Relationship between qSPCB10.1 and qOCA08.1 in SPC and SOC Accumulation

**Figure 4:**
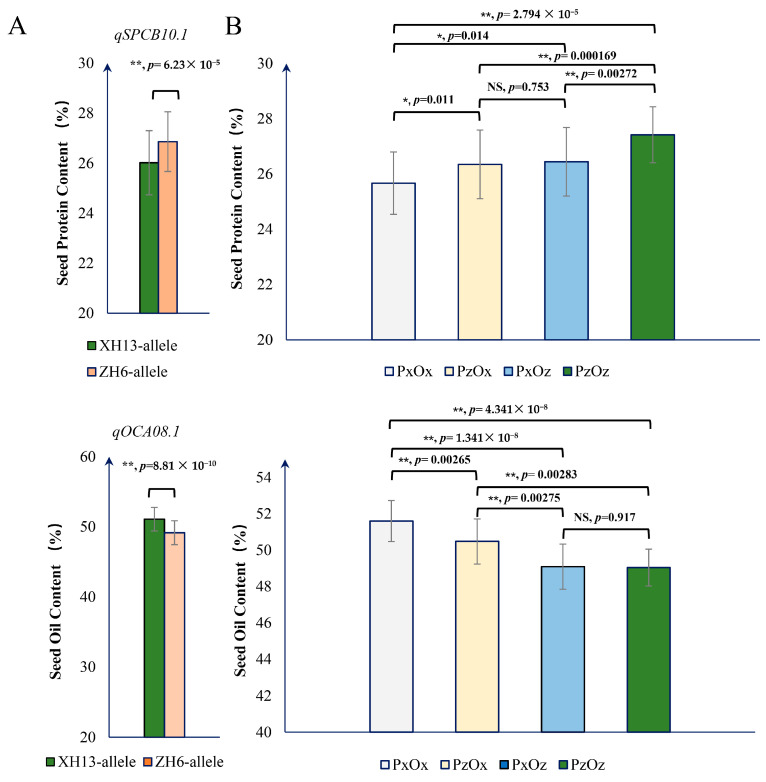
Interactions between *qSPCB10.1* and *qOCA08.1* and their effects on peanut SPC and SOC. (**A**) The effect of different alleles of *qSPCB10.1* and *qOCA08.1* on SPC and SOC, respectively. Error bars represent the SE values; statistical significance was determined by an unpaired t-test; “**” represents *p* < 0.01. (**B**) Interactions between various *qSPCB10.1* and *qOCA08.1* allele combinations in SPC and SOC. “P” represents *the qSPCB10.1* locus, and “O” represents the *qOCA08.1* locus. “x” represents the allele from XH13, while “z” represents the allele from ZH6. Error bars represent the SE values, and statistical significance was determined by unpaired t-test. “**”, “*”, and “NS” represent *p* < 0.01, *p* < 0.05, and no significant difference, respectively.

In peanut, significant and negative correlations between seed protein content and oil content have been reported in many studies. In a previous study, Liu et al. identified *qOCA08.1*, one major QTL controlling seed oil content in chromosome 8, using an RIL population derived from ZH6 and XH13, the same parents used in this study [11]. Hence, we analyzed the effects and putative relationship between *qSPCB10.1* and *qOCA08.1* in regulating the seed protein and oil contents in peanut. The marker PCB10.1 was developed and used to detect the presence of the *qSPCB10.1* allele in the tested RIL population. In contrast, the marker SNPO08.1 developed in a previous study was used to detect the *qOCA08.1* allele. As expected, lines containing the ZH6 allele of *qSPCB10.1* (*qSPCB10.1_ZH6_*) exhibited a significantly higher SPC (*p* = 6.23 × 10^−5^) than lines carrying the XH13 allele of *qSPCB10.1* (*qSPCB10.1_XH13_*). In contrast, lines carrying the XH13 allele of *qOCA08.1* (*qOCA08.1_XH13_*) exhibited a significantly higher SOC (*p* = 8.81 × 10^−10^) than lines with the ZH6 allele of *qOCA08.1* (*qOCA08.1_ZH6_*). This suggests that both of these loci have distinct roles in peanut seed protein and seed oil content, respectively (Figure 4A).

Interestingly, it was found that *qSPCB10.1_ZH6_/qOCA08.1_ZH6_* (P_Z_O_Z_) lines had a significantly higher SPC than *qSPCB10.1_ZH6_/qOCA08.1_XH13_* (P_X_O_Z_) lines (*p* = 0.00272). On the contrary, *qSPCB10.1_XH13_/qOCA08.1_ZH6_* (P_Z_O_X_) lines had a higher SPC (*p* = 0.011) than lines with the *qSPCB10.1_XH13_/qOCA08.1_XH13_* (P_X_O_X_) genotype. Meanwhile, *qSPCB10.1_ZH6_/qOCA08.1_ZH6_* (P_Z_O_Z_) lines exhibited a significantly higher SPC than *qSPCB10.1_ZH6_/qOCA08.1_XH13_* (P_Z_O_X_) lines (*p* = 0.000169), while *qSPCB10.1_XH13_/qOCA08.1_ZH6_* (P_X_O_Z_) lines exhibited a higher SPC than lines carrying the *qSPCB10.1_XH13_/qOCA08.1_XH13_* (P_X_O_X_) alleles (*p* = 0.014). These results suggest that *qOCA08.1* negatively affects seed protein accumulation regardless of the presence of *qSPCB10.1*.

We also compared SOC in genotypes with different allelic combinations. The lines with the *qSPCB10.1_XH13_/qOCA08.1_XH13_* (P_X_O_X_) genotype showed a significantly higher SOC (*p* = 0.00265) than that of lines with the *qSPCB10.1_ZH6_/qOCA08.1_XH13_* genotype (P_Z_O_X_). In comparison, *qSPCB10.1_XH13_/qOCA08.1_ZH6_* (P_X_O_Z_) lines did not exhibit a significantly higher SOC than the *qSPCB10.1_ZH6_/qOCA08.1_ZH6_* (P_Z_O_Z_) lines (*p* = 0.917), suggesting *qSPCB10.1* may play a negative role in seed oil accumulation in the presence of the *qOCA08.1_XH13_* allele. However, *qSPCB10.1* did not influence SOC in the presence of *qOCA08.1_ZH6_* (Figure 4B).

## 3. Discussion

Population growth and food crises compel an increase in protein production. Peanuts, a protein-rich plant, have the highest protein content among nuts consumed daily [12]. Hence, it is promising to produce more plant-based protein by elevating the seed protein content in peanuts. However, unlike the SOC in peanuts, the SPC has not received sufficient attention in genetic studies. Few QTLs controlling SPC have been identified, and the genetic mechanism underlying SPC is still obscure, limiting breeding programs for high-protein peanut varieties. Consequently, it is imperative that we identify additional genetic loci that regulate SPC in peanuts.

In this study, *qSPCB10.1*, a locus underlying seed protein content in peanuts, was narrowed down to a 2.3 Mb genomic region by QTL-seq analysis. QTL-Seq is a powerful technique when combined with bulked segregant analysis (BSA) and high-throughput whole-genome re-sequencing, enabling the detection of major loci controlling agronomic traits [13]. In peanut, QTL-seq has been applied to identify genetic loci controlling important agronomic traits, both for qualitative traits controlled via one or few loci, such as testa color [14,15], kernel sucrose content [16], and biotic stress resistance [17,18], and for complex traits influenced via many genetic factors, such as seed size [19], seed shell percentage [20], and seed dormancy [21]. Previous studies on SPC in peanut have reported no QTLs underlying SPC in the B10 region [8,9,10], indicating that *qSPCB10.1* is a new major locus controlling SPC in peanut.

There were 494 SNPs/InDels variants and 63 predicted genes in the *qSPCB10.1* region. However, no variants in the mapped region resulted in frame-shift, stop-gain/loss, or splicing variations in these predicted genes. In addition, two predicted genes that harbored non-synonymous mutations caused by SNPs/InDels variants did not have detectable expression. These findings suggested that *qSPCB10.1* may not exert its function through a structural gene variant. In the *qSPCB10.1* region, seven DEGs were identified based on the transcriptomic data from the developing seed. The possibility of a candidate gene was ruled out for *arahy.533ER3*, as no sequence variations between two parents were detected in its gene body and the surrounding region. For the remaining six predicted genes, *arahy.GFBT7E* was annotated as an oleosin. Oleosin accounts for 8% of the total seed protein and 80–90% of the oil body proteins [22]. It plays an important role in lipid storage [23]. The temporal expression patterns of the oleosin genes in maturing seeds have been reported to be similar to those of seed storage proteins [24]. Although there is no evidence of the direct relationship between the amount of oleosin and seed oil/protein content, it may serve as the recognition signal for the specific binding of lipase to lipid bodies in the lipid degradation pathway [25]. *arahy.A6X2CD* encodes a serine/threonine protein kinase. In previous studies, serine/threonine protein kinases have been reported to be associated with seed development and regulation of seed size [26]. In addition, they may affect SOC via participating in histone modifications, which can alter oil content [27]. Interestingly, a serine/threonine/tyrosine protein kinase has been reported to phosphorylate oleosin in peanut seed [28]. Hence, combined with sequence analysis, transcriptomic data, and functional annotation data, we proposed that *arahy.GFBT7E* and *arahy.A6X2CD* may probably be the candidate genes for *qSPCB10.1*.

SPC is a complex quantitative trait governed by multiple genetic factors during seed development. Generally, the genes underlying SPC can be classified into two types. The ones that are pleiotropic often simultaneously influence SPC, seed size, and SOC. Interestingly, under the regulation of these genes, such as *POWR1*, *GmSWEET10a*, *GmSWEET10b*, and *GmST05* in soybean, the SPC exhibited a significant negative correlation with SOC [29,30,31]. It has been reported that larger-sized seeds typically accumulate a higher amount of oil content [32]. As the carbon sources in seeds are taken up to synthesize more oil, the carbon sources for storage protein are limited, and the SPC is consequently reduced. On the other hand, there are genes that specifically regulate SPC without influencing other traits. These genes often influence the biosynthesis and transportation of amino acids or storage proteins. For example, *TEOSINTE HIGH PROTEIN 9* (*THP9*), encoding an asparagine synthetase enzyme, is highly expressed in teosinte and significantly increases SPC in maize [33]. In soybean, *GmRab5a* and its guanine exchange factors *Gm*VPS9s regulate SPC by influencing the post-Golgi trafficking of storage proteins [34].

The SPC-specific QTLs are more desirable in breeding programs because they enable the selection of favorable lines without affecting other important agronomic traits, such as SOC or seed size. In this study, we identified *qSPCB10.1* underlying SPC. Our data analysis revealed that the *qSPCB10.1* gene alone does not have an impact on SOC unless it is present simultaneously with the *qOCA08.1* allele from the high-SOC parent XH13. This result was further validated by a QTL analysis in a previous study, as no QTL underlying SOC was identified in the *qSPCB10.1* region [11]. However, we found that the XH13 allele from *qOCA08.1* itself can influence SPC without an interaction from *qSPCB10.1*, while *qOCA08.1_ZH6_* could not, suggesting *qOCA08.1* may have a pleiotropic effect. This relationship should be addressed when screening high-SPC peanut lines by marker-assisted selection breeding.

## 4. Materials and Methods

### 4.1. Plant Material

“Zhonghua 6” (ZH6) is an elite peanut cultivar with a relatively higher SPC that was developed by the Oil Crops Research Institute of the Chinese Academy of Agricultural Sciences. “Xuhua 13” (XH13) is an elite intermediate-type peanut variety with a relatively lower SPC, released via the Xuzhou Institute of Agricultural Sciences. An RIL population consisting of 160 lines was constructed and analyzed in our study using XH13 as the female parent and ZH6 as the male parent.

### 4.2. Phenotypic Evaluation

The parental lines and the RIL population were planted in the same experimental field in Nanping City, Fujian Province, and Wuhan City, Hubei Province, China. The parental lines were also planted in Quanzhou City, Fujian Province, China. The field experiments were performed in a randomized complete block design with three replications. Each experimental plot contained 10 plants. The plant density and field management followed local agricultural management practices.

The SPC was measured by NIR spectroscopy on a PerkinElmer DA 7250 diode array NIR system using the near-infrared spectroscopy method. The matured seeds (those plump seeds harvested from pods with brown or black pod mesocarp color [35]) with less than 10% moisture content were used for analysis. The SPC was determined by the average result of three parallel measurements on each sample. The SOC was measured using the near-infrared spectroscopy method. The standards for evaluating the tested seeds were similar to those of SPC determination.

### 4.3. Preparation of DNA Bulks and Illumina Sequencing

Based on the SPC phenotypes from the two trial sites, the young leaves from the parental lines ZH6 and XH13, 25 RILs with a high SPC and 25 RILs with a low SPC were collected and used for genomic DNA extraction following a modified CTAB method. The quality and concentration of each DNA sample was analyzed using 1% agarose gel electrophoresis and NanoDrop. The LSB pool and HSB pool were generated by mixing equal amounts of total DNA from each collected LSB and HSB DNA sample. The DNA libraries (including LSB, HSB, and the parental lines ZH6 and XH13) were constructed following the protocol of the NEBNext Ultra II DNA Library Prep Kit for Illumina. High-throughput sequencing of the DNA libraries was performed using the Illumina NovaSeq platform with the NovaSeq 6000 S4 Reagent Kit in Genoseq Technology Co., Ltd. (Wuhan, China).

### 4.4. Variant Detection

The variant detection pipeline was described in a previous study [36]. Cutadapt and Trimmomatic were used to obtain high-quality clean data from the Illumina sequencing data by removing ineligible reads, such as low-quality reads (Q < 20), adapter sequences, N > 10% reads, and too-short reads (<20 bp). The high-quality clean data were aligned to a reference cultivated peanut genome (https://data.legumeinfo.org/Arachis/hypogaea/genomes/Tifrunner.gnm2.J5K5/, accessed on 5 December 2022) using the BWA software (0.7.17) [37]. After obtaining the alignment result, the duplicates and the PCR repeats were removed by “SortSam” in “Picard” tools. Variant detection was performed by the “HaplotypeCaller” module in “GATK” [38], and ANNOVAR was used to annotate those variants [39].

### 4.5. BSA-Seq Analysis

BSA-seq analysis was performed using the QTLseqr R package [40]. The genomic region underlying SPC was determined by the Δ (SNP index) between genomic regions from LSB and HSB DNA pools [13]. In this study, the variants with a Δ (SNP index) value were determined at a significance level of *p* < 0.01.

### 4.6. RNA-Sequencing and Gene Expression Profile Analysis in the Mapped Region

Total RNA was extracted from the developing seeds of ZH6 and XH13 (with three biological replications for each parental line) at 60 days after flowering using a TRIzol^®^ reagent kit (Invitrogen, Carlsbad, CA, USA) according to the manufacturer’s protocol. RNA concentration, purity, and integrity were evaluated using an Agilent 2100 Bioanalyzer (Agilent Technologies, Palo Alto, CA, USA) and 1% agarose gel electrophoresis. The libraries were constructed using the TruSeq Stranded kit (Illumina, San Diego, CA, USA) following the manufacturer’s instructions. Sequencing (MiSeq Reagent Kit v3, 150 cycles) was performed on an Illumina MiSeq sequencer in Beijing Biomarker Technologies Co., Ltd. Reads were mapped to the cultivated peanut reference genome (https://data.legumeinfo.org/Arachis/hypogaea/genomes/Tifrunner.gnm2.J5K5/, accessed on 5 December 2022) using HISAT2. Fragments per kilo-base of transcript per million (FPKM) were estimated to quantify the gene expression levels [41]. The differentially expressed genes (DEGs) were analyzed through DESEeq2 [42]. Multiple hypotheses with the p-value thresholds of fold change (FC) ≥ 2 and false discovery rate (FDR) ≤ 0.05 were applied. Genes with FPKM > 0.5 based on our RNA-seq data were considered as “with obvious expressional profile” in this study.

### 4.7. Marker Development

Newly developed SNP markers used for mapping and genotyping were designed based on the re-sequencing analysis of two parental lines (ZH6 and XH13). All these SNP markers were developed using the procedure described in the previous study [43]. The sequence information of all primers used in this study is listed in Table S4.

## Data Availability

Data are contained within the article and Supplementary Materials.

## Supplementary Materials

The following supporting information can be downloaded at: https://www.mdpi.com/article/10.3390/plants13172368/s1: Table S1, annotation of predicted genes in *qSPCB10.1* mapping region; Table S2, SNPs in putative candidate genes in the mapping genomic region on chromosome B10; Table S3, InDels in putative candidate genes in the mapping genomic region on chromosome B10; Table S4, primers used in this study.
